# Engineering *Comamonas testosteroni* for the production of 2-pyrone-4,6-dicarboxylic acid as a promising building block

**DOI:** 10.1186/s12934-023-02202-2

**Published:** 2023-09-19

**Authors:** Tom Delmulle, Stijn Bovijn, Sari Deketelaere, Martijn Castelein, Tom Erauw, Matthias D’hooghe, Wim K. Soetaert

**Affiliations:** 1 Centre for Industrial Biotechnology and Biocatalysis (InBio.be), Department of Biotechnology, Faculty of Bioscience Engineering, Ghent University, Coupure Links 653, Ghent, 9000 Belgium; 2 SynBioC Research group, Department of Green Chemistry and Technology, Faculty of Bioscience Engineering, Ghent University, Coupure Links 653, Ghent, 9000 Belgium

**Keywords:** 2-pyrone-4,6-dicarboxylic acid, Upcycling, *Comamonas*, Terephthalic acid

## Abstract

**Background:**

Plastics are an indispensable part of our daily life. However, mismanagement at their end-of-life results in severe environmental consequences. The microbial conversion of these polymers into new value-added products offers a promising alternative. In this study, we engineered the soil-bacterium *Comamonas testosteroni KF-1*, a natural degrader of terephthalic acid, for the conversion of the latter to the high-value product 2-pyrone-4,6-dicarboxylic acid.

**Results:**

In order to convert terephthalic acid to 2-pyrone-4,6-dicarboxylic acid, we deleted the native PDC hydrolase and observed only a limited amount of product formation. To test whether this was the result of an inhibition of terephthalic acid uptake by the carbon source for growth (i.e. glycolic acid), the consumption of both carbon sources was monitored in the wild-type strain. Both carbon sources were consumed at the same time, indicating that catabolite repression was not the case. Next, we investigated if the activity of pathway enzymes remained the same in the wild-type and mutant strain. Here again, no statistical differences could be observed. Finally, we hypothesized that the presence of a *pmdK* variant in the degradation operon could be responsible for the observed phenotype and created a double deletion mutant strain. This newly created strain accumulated PDC to a larger extent and again consumed both carbon sources. The double deletion strain was then used in a bioreactor experiment, leading to the accumulation of 6.5 g/L of product in 24 h with an overall productivity of 0.27 g/L/h.

**Conclusions:**

This study shows the production of the chemical building block 2-pyrone-4,6-dicarboxylic acid from terephthalic acid through an engineered *C. testosteroni KF-1* strain. It was observed that both a deletion of the native PDC hydrolase as well as a *pmdK* variant is needed to achieve high conversion yields. A product titer of 6.5 g/L in 24 h with an overall productivity of 0.27 g/L/h was achieved.

**Supplementary Information:**

The online version contains supplementary material available at 10.1186/s12934-023-02202-2.

## Background

It is hard to imagine a world without plastics as they are used in almost every perceivable application in our everyday lives. Unfortunately, most fossil-derived plastics are not readily degradable and lead to ever-increasing severe environmental impacts. With 76% of all plastics being landfilled or discarded, often after a service life of less than one year, an estimated 20 Mton end up in the oceans every year [1]. Indeed, by 2050, it is estimated that 1.1 Gton of plastic will be present in the environment [2]. Although regulations are being put in place to reduce plastic usage, there is still a clear need for more sustainable end-of-life solutions. In this regard, the microbial upcycling of plastic waste streams offers a promising alternative. In this approach, plastics are enzymatically or chemically degraded into smaller components which can then be extracted and used as building blocks for other components with a higher value [3]. In recent years, many enzymes capable of degrading various types of plastics, including polyethylene terephthalate (PET), polyurethane (PUR), polyethylene (PE), and polypropylene (PP) have been identified and engineered to improve activity [3–5]. Also from the downstream steps of converting the obtained components into higher value-added products, a lot of research has been performed. Since one of the first plastic degrading enzymes to be identified was active on PET, the vast majority of research has been on the microbial upcycling of PET monomers. Indeed, the production of a wide variety of added-value metabolites from PET monomers have already been reported including, but not limited to, vanillic acid, gallic acid, pyrogallol, muconic acid, glycolic acid, polyhydroxyalkanoates, catechol, and 2-pyrone-4,6-dicarboxylic acid (PDC) [6–12].

PDC is an intermediate in metabolic pathways like the protocatechuate 4,5-cleavage pathway involved in lignin degradation [13]. It is a chemical that is not being synthesized chemically but has several interesting properties thanks to its pyrone ring and the two carboxylic acid moieties [14]. Polyesters of PDC and 1,4-butanediol, for example, showed high thermal stability, as it was suggested that crosslinking with the ester in the ring also took place [15]. Polyesters with 1,2-ethanediol, 1,3-propanediol or bis(hydroxyethyl)terephthalate again had high thermal stability as well as a high Young’s modulus and showed 60 to 100% degradation in less than 3 weeks [16]. Bis(hydroxyethyl) PDC/TPA polyesters displayed strong adhesion to metal surfaces as well [16]. The increased biodegradability is attributed to the ring opening of PDC and was also observed in a copolymer with polybutylene succinate [17]. Next to synthesis of polymers, other applications are also possible like, for example, the preferential trapping of radioactive cesium ions in contaminated water [18, 19]. Due to its interesting properties, the microbial production of PDC has been the focus of many research efforts in the past years [6, 15, 20–25].

In this study, we studied the use of the soil-bacterium *Comamonas testosteroni KF-1*, a strain capable of naturally consuming TPA, for the upcycling of TPA to the value-added product PDC. To do so, we created a mutant with a deletion in the natural TPA degradation pathway so that PDC could be accumulated. This conversion was further optimised through the deletion of an additional gene and the PDC production capacity was finally evaluated in a fed-batch process.

## Results and discussion

### Engineering *C. testosteroni KF-1* for the production of PDC from TPA

**Fig. 1:**
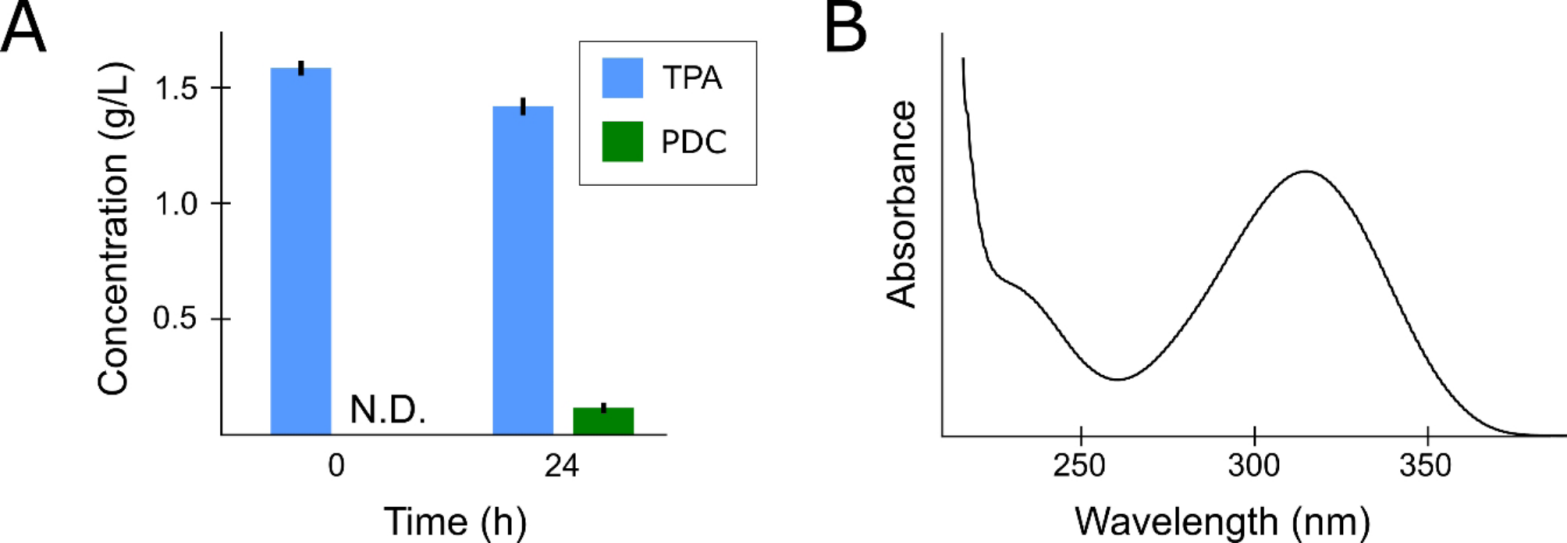
Production of PDC by sCT01 from TPA and glycolic acid. (**A**) Concentration of TPA and PDC at the start of the experiment (0 h) and end of the experiment (24 h). (**B**) Absorption spectrum of the newly produced PDC in 5 mM H_2_SO_4_. N.D.: Not detected

The gram-negative bacterium *C. testosteroni KF-1* is a natural degrader of TPA [26]. It does so through conversion to protocatechuic acid (PCA) which is further assimilated to intermediates of the central carbon metabolism. Within this degradation route, PDC is a natural intermediate that is formed by the dehydrogenation of 4-carboxy-2-hydroxymuconate-6-semi-aldehyde. In order to accumulate PDC from TPA and prevent its further consumption, further hydrolysis of the former was blocked through the deletion of the corresponding PDC hydrolase present in the PCA degradation operon of *C. testosteroni KF-1*. The resulting strain, i.e. sCT01, was then cultivated on a minimal medium containing TPA and glycolic acid as carbon sources. While the former should be converted to PDC, the latter served as a carbon source for growth. However, after 24 h of growth no statistical amounts of TPA were consumed by the strain (p-value 0.172) and only 120 ± 45 mg/L of a new product of which the mass and UV-spectrum corresponded to PDC was detected [27] (Fig. 1, A and B). The latter is in strong contrast with other published research in which an engineered *Escherichia coli* strain was capable of converting TPA to PDC with a 91% molar yield [6].

**Fig. 2:**
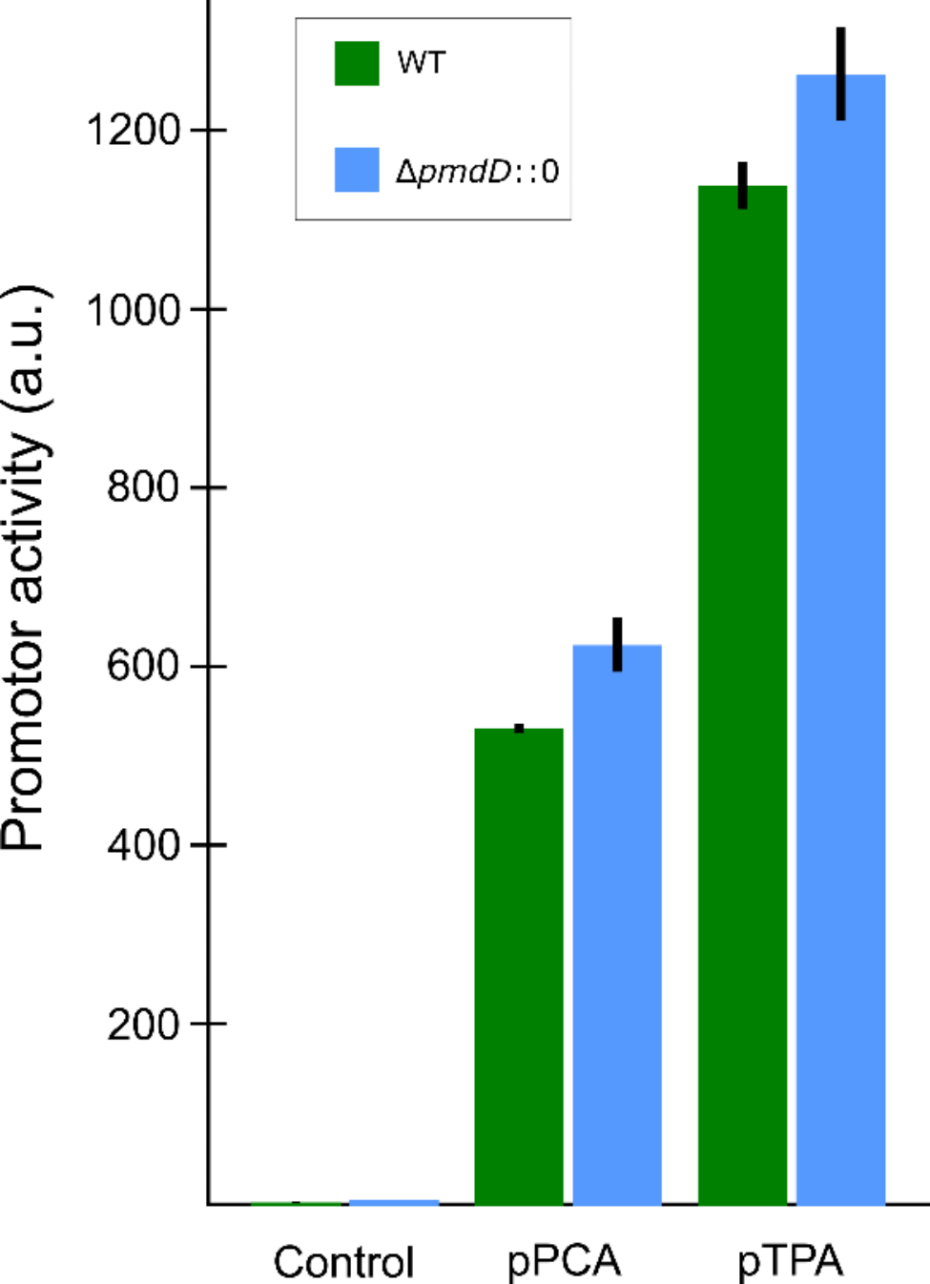
Measured activity of the promoter regions upstream of the PCA and TPA degradation operons. Activity was induced through the addition of TPA to the medium. Promoter activity was tested both in the wild-type strain (*C. testosteroni KF-1*) as well as the strain with a deletion of *pmdD* (sCT01)

The lack of TPA consumption is in strong contrast with the observation that *C. testosteroni KF-1* is capable of using TPA as the sole carbon source for growth (Weiss et al. 2013 and this study). To investigate whether the presence of glycolic acid led to an inhibition in TPA consumption due to the preferential consumption of the former, we cultivated the wild-type strain in the same minimal medium and monitored TPA and glycolic acid consumption over time. In contrast to strain sCT01, the wild-type assimilated both glycolic acid and TPA at the same time showing that substrate preference is not the cause for the limited PDC production and TPA consumption in strain sCT01 (Supplementary Figure S1). Having shown that both TPA and glycolic acid are consumed at the same time, we next investigated if the operons responsible for the conversion of TPA to PCA and PCA to PDC were still expressed and were not inhibited by the small amounts of PDC that were produced. To do so, the upstream regions of these operons were amplified and cloned in front of a fluorescent protein to measure protein activity indirectly. The corresponding plasmids, containing either the upstream region of the TPA degradation operon or the PCA degradation operon (pCT07 and pCT08, respectively), were transformed both in the wild-type as well as the deletion strain to compare promoter activity. The corresponding strains were cultivated in a minimal medium containing glycolic acid as the carbon source and fluorescence was induced through the addition of TPA. Unfortunately, no statistical difference in promoter strength could be detected between the wild-type and the deletion strain for both operons (Fig. 2).

**Fig. 3:**
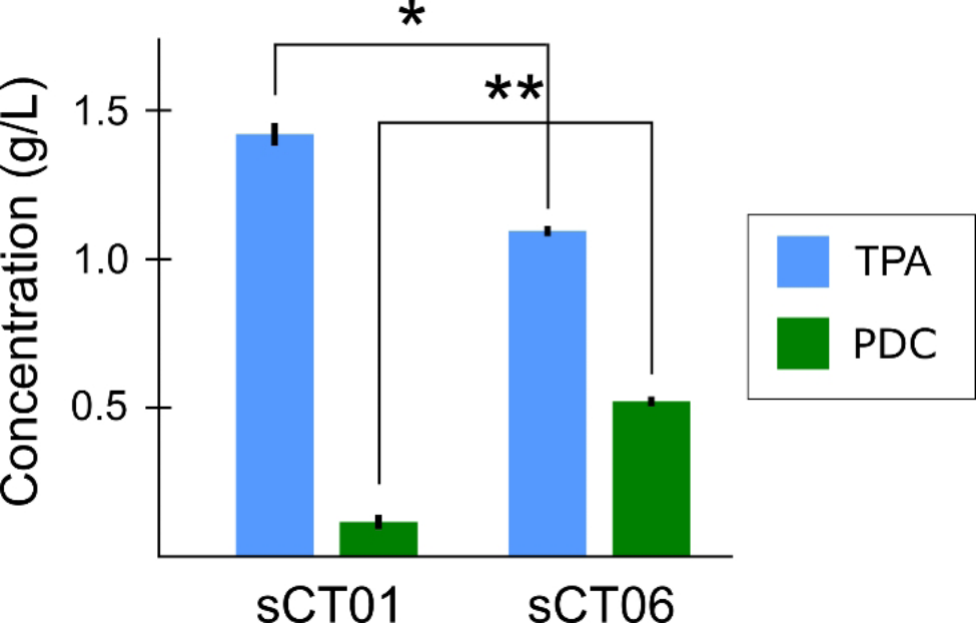
Production of PDC by sCT01 and sCT06 from TPA and glycolic acid. * p-value < 0.05, ** p-value < 0.01

With a wild-type strain capable of co-consuming TPA and glycolic acid, and with a similar promoter activity in both the wild-type and deletion strain, it was clear that the mechanism by which the deletion strain is seemingly prevented of assimilating TPA was more complex. A reduction of TPA uptake has already been reported in *C. testosteroni* sp strain E6 when PCA is present in the medium [28]. However, this only resulted in a reduction of TPA uptake while our results indicate an almost complete inhibition. Furthermore, no detectable amounts of PCA could be observed in the deletion strain. On the other hand, a recent study indicated that PDC is a more potent inducer of the PCA operon than PCA [29]. By consequence, small amounts of PDC can have a high inducing effect on promoter activity and can lead to greater transcription rates. Furthermore, the research on the inhibiting effect of PCA on TPA uptake mentions a *pmdK* variant encoding a 4-hydroxybenzoate transporter present in *C. testosteroni* which could attribute to the observed phenotype [28]. Interestingly, this *pmdK* variant is part of the PCA degrading operon and could thus be greatly induced by PDC. Indeed, when grown on aromatic carbon sources such as 4-hydroxybenzoic acid, vanillate, and TPA, this gene was found to be highly induced in *C. testosteroni KF-1* [30]. We therefore hypothesised that the uptake of TPA was inhibited through the increased expression of the *pmdK* variant as a consequence of the accumulation of PDC and hence induction of the PCA degradation operon by PDC. To test this hypothesis, we deleted the *pmdK* variant gene in our deletion strain and retested the capacity for PDC production on minimal medium containing both TPA and glycolic acid. Significantly more TPA was consumed (p-value 0.018) and the double deletion strain accumulated significantly more of the presumed PDC product (524 ± 32 mg/L, p-value 0.002, Fig. 3). Yet, still a large amount of TPA remained at the end of the production trial, reducing the overall yield. Nevertheless, PDC could be produced to an amount allowing confirmation of its molecular structure via NMR spectra (Supplementary Figure S2).

The exact mechanism by which *pmdK* leads to reduced TPA uptake is however still unclear. One hypothesis is that the increased expression of *pmdK* leads to a reduced or even inhibited interaction between the periplasmic TPA solute binding protein and the transporter complex encoded by the *TpiAB* genes. The latter interaction is crucial for TPA uptake as shown in a recent study [28]. The reduced uptake by increased expression of the *pmdK* gene could lead to enzyme crowding on the cell surface and could thus hinder the formation of a functional transporter complex for TPA. However, this hypothesis would need further investigation.

### Production of PDC at bioreactor scale

**Fig. 4:**
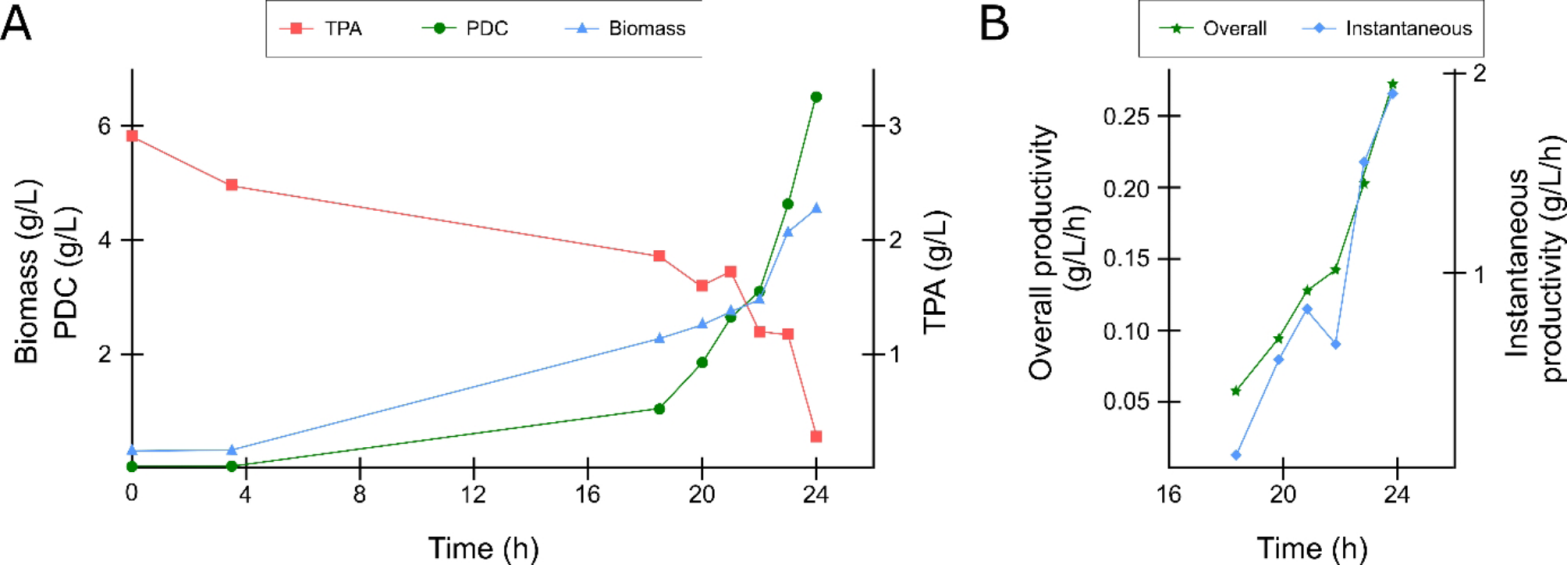
Production of PDC at bioreactor scale from TPA and glycolic acid using strain sCT06. (**A**) Time profile of the concentration of TPA, PDC and biomass. (**B**) Overview of the overall productivity and instantaneous productivity over time

After successfully showing PDC production with the double deletion strain on shake flasks, we investigated the capabilities of this strain in a bioreactor experiment to further boost product titers and productivities. To do so, a fed-batch operation was pursued in which the increase in pH due to the consumption of glycolic acid was used as a measure to add fresh glycolic acid to the fermentation broth. This pH-stat mode of operation has recently also been successfully used for the production of PHB from acetic acid [31]. Furthermore, next to the feeding of glycolic acid, also TPA was fed to increase product titers while avoiding potential inhibition due to a high aromatic content. This approach resulted in the production of 6.5 g/L PDC with an overall productivity of 0.27 g/L/h (Fig. 4). Interestingly, the instantaneous productivity (i.e. the productivity between samples) increased over time and reached a value of 1.87 g/L/h in the final hour (Fig. 4, B). The results obtained within this study are comparable to the results obtained in previous studies (Table 1).

**Table 1:** Comparison of the PDC titer and productivity obtained in this study with previously reported values. *: Estimated from figures

Strain	Carbon source	PDC product titer (g/L)	PDC productivity (g/L/h)	Reference
*Comamonas testosteroni KF-1*	Terephthalic acid	6.5	0.27	This study
*Escherichia coli XL1-Blue*	Terephthalic acid	0.58	0.097	(6)
*Pseudomonas putida KT2440*	4-hydroxybenzoate	58.0	0.2	(20)
*Pseudomonas putida KT2440*	p-coumaric acid	22.7	0.21	(15)
*Pseudomonas putida PpY1100*	Protocatechuic acid	± 11 *	± 0.31 *	(21)
*Pseudomonas putida PpY1100*	Vanillic acid	99.9	1.69	(22)
*Pseudomonas putida PpY1100*	Lignin	9.4	0.39	(23)
*Escherichia coli W3110*	Glucose	16.72	0.172	(24)
*Escherichia coli W3110*	Glucose	0.17	0.003	(25)

## Conclusions

In this study we showed the production of PDC from TPA and glycolic acid as carbon sources. To produce PDC, we created a knock-out of the natively present PDC hydrolase to channel all the provided TPA to PDC. However, only a limited amount of PDC was produced and only a small fraction of the provided TPA was consumed at this stage. To solve this issue, we investigated the co-consumption of the different carbon sources and evaluated the promoter activity of the different operons involved in conversion of TPA to PDC. The co-consumption of TPA and glycolic acid was confirmed and no statistical difference in promoter activity between the wild-type and knock-out strain could be detected. We therefore hypothesised that the presence of the *pmdK* gene in the PCA degradation operon hindered the production capabilities of our strain. Deletion of the former improved product titers and solved the issue of limited TPA consumption. This newly created double knock-out strain was then evaluated in a bioreactor resulting in 6.5 g/L PDC in 24 h with an overall productivity of 0.27 g/L/h. This research thus forms the basis for further strain engineering towards the upcycling of PET monomers into value-added products.

## Methods

### Chemicals, oligonucleotides and standard molecular techniques

Unless otherwise stated, all products were purchased from Sigma Aldrich (Overijse, Belgium), product concentration in % are w/v, PCR products were purified by the innuPrep PCRpure Kit (Analytik Jena AG, Jena, Germany) and plasmid extraction was performed with the Qiagen Spin Miniprep kit (Qiagen, Antwerp, Belgium). Oligonucleotides were obtained from Integrated DNA Technologies (IDT, Leuven, Belgium) and sequencing was performed by EZ-Seq (Macrogen, Amsterdam, The Netherlands). In case linear DNA fragments were needed for further cloning, they were amplified using the high-fidelity PrimeSTAR HS DNA polymerase (Takara, Westburg, The Netherlands, R040A), according to the manufacturers’ instructions. Colony PCR to confirm plasmid assembly or transformations were done using Taq DNA polymerase (NEB, M0273X) according to the manufacturers’ instructions.

## Strains and media

One Shot TOP10 chemically competent™ *Escherichia coli* (C404003, ThermoFisher Scientific, Aalst, Belgium) was used for cloning procedures and to maintain plasmids. *E. coli* strains were cultured using Lysogeny Broth (LB) consisting of 10 g/L tryptone (BD Difco), 5 g/L yeast extract (BD Difco) and 5 g/L sodium chloride (VWR, Leuven, Belgium). 12 g/L agar (Biokar Diagnostics, Pantin Cedex, France) was added if solid medium was required. All components were autoclaved at 121 °C for 21 min (PriorClave, London, United Kingdom). To maintain plasmids, the required antibiotic, 100 µg/mL ampicillin (filter-sterilised), 50 µg/mL kanamycin (filter-sterilised), 25 µg/mL chloramphenicol (filter-sterilised) or 10 µg/mL tetracycline (filter-sterilised), was added after autoclaving. Cultures were incubated overnight at 37 °C, while shaking at 200 rpm in the case of liquid cultures. 70% glycerol in a 1:1 ratio mixture was used to store *E. coli* strains in cryotubes at -80 °C.

*Comamonas testosteroni KF-1* (DSM 14,567, Leibniz-Institut DSMZ) was used as the basis for further strain engineering. For routine handling, *C. testosteroni* was cultured using LB. To maintain plasmids or to select for genomic integration events, the required antibiotic, 50 µg/mL chloramphenicol or 10 µg/mL tetracycline, was added. Cultures were incubated at 30 °C, while shaking at 200 rpm in the case of liquid cultures. 70% glycerol in a 1:1 ratio mixture was used to store *C. testosteroni* strains in cryotubes at -80 °C.

In the case *C. testosteroni* strains were used for production or growth experiments, a minimal medium was used for which the pH was adjusted using 5 M KOH or 5 M HCl (Table 2). Both the carbon and nitrogen source were autoclaved separately from the other medium components. After autoclaving, a solution of trace elements was added (Supplementary Table S1). In all cases a C:N ratio of 10 was used.

**Table 2:** Composition of the minimal medium used in this study. A solution of trace elements was added after autoclaving (Supplementary Table S1)

Component	Concentration/Value
Carbon source:	
- TPA - Glycolic acid	10 mM 25 mM
(NH_4_)_2_SO_4_	Adjusted to C:N ratio of 10
MgCl_2_.7H_2_O	0.5 g/L
Phosphate buffer	75 mM
pH	7

### Plasmid construction

The different plasmids used in this study (Table 3) were constructed via Golden Gate Assembly by incorporating restriction sites into the primers that were used to amplify the different genetic parts. Suicide plasmids were used to construct knock-out strains and consisted of a pUC ori, a tetracycline resistance marker and the homologous regions (1000 bp) flanking the gene to be deleted. The tetracycline marker was cloned in between the homologous regions and provided selective pressure to select for knock-out strains. Furthermore, suicide plasmids also contained an I-SceI recognition site to remove the marker from the genome in a second round of transformation. To do so, a recovery plasmid was constructed containing the same homologous regions fused together as well as an expression cassette for the codon-harmonised I-SceI gene. The latter consisted of the upstream region of the TPA degradation operon and had a theophylline-dependent riboswitch downstream of the I-SceI coding sequence to control its expression [32]. The recovery plasmid itself consisted of a pBBR1 backbone in which the kanamycin resistance marker was replaced by a chloramphenicol resistance marker. The same plasmid backbone was also used for the construction of the expression plasmids used in the determination of the promoter activity. In short, the intergenic upstream region in front of the TPA and PCA degradation operon were amplified and cloned in front of the mCherry fluorescent protein.

**Table 3:** Overview of the different plasmids used in this study

Plasmid ID	Plasmid content	Reference
pBBR1MCS-2	Host plasmid with Km^r^ and pBBR1 ori	[33]
pCT01	Suicide plasmid with Tet^r^ and pUC ori	This study
pCT02	pCT01, *pmdD* deletion vector	This study
pCT03	Host plasmid with Chlor^r^ and pBBR1 ori	This study
pCT04	pCT03 with *P*_*TPA*_, *I-SceI*	This study
pCT05	pCT04 with *pmdD* recovery cassette	This study
pCT06	pCT04 with *pmdD* and *pmdK* recovery cassette	This study
pCT07	pCT03 with *P*_*TPA*_, *mCherry*	This study
pCT08	pCT03 with *P*_*PCA*_, *mCherry*	This study

### Transformation of *C. testosteroni* and creation of genomic deletions

Suicide and expression plasmid were transformed in *C. testosteroni KF-1* as described earlier [34]. In short, an overnight pre-culture grown in LB medium was used to inoculate 25 mL of fresh LB medium and was incubated at 30 °C for 3 h. The culture was then chilled on ice, centrifuged and washed 3 times with 10% sterile, ice-cold glycerol solution before being resuspended in 50 µL of the latter. To this cell solution, 2 µL of the desired plasmid was added and the suspension was electroporated using a chilled 2-mm electroporation cuvette (Bio-Rad) at 2.5 kV. One mL of LB was added immediately after electroporation and the suspension was resuscitated at 30 °C for 2 h before being plated on selective LB plates.

Genomic deletions were constructed via a suicide vector-based method. In a first step, a suicide plasmid containing a tetracycline resistance marker in between the flanking homologous regions was transformed in the desired *C. testosteroni* strain and successful integration of the plasmid was selected for on tetracycline containing LB plates. After confirmation via PCR, a successful colony was retransformed with the recovery plasmid and selected for on chloramphenicol containing plates. The leaky expression of the I-SceI expression cassette proved sufficient for immediate recovery of the tetracycline marker from the genome. To remove the recovery plasmids, cultures were subcultivated in LB medium and the loss of plasmid was confirmed through antibiotic sensitivity tests.

A list of all strains used in this study can be found in Table 4.

**Table 4:** Overview of the different strains used in this study

Strain ID	Genotype	Reference
*Comamonas testosteroni KF-1*	Wild-type	[26]
sCT01	*C. testosteroni KF-1* Δ*pmdD*::0	This study
sCT02	*C. testosteroni KF-1* + pCT07	This study
sCT03	*C. testosteroni KF-1* + pCT08	This study
sCT04	*C. testosteroni KF-1* Δ*pmdD*::0 + pCT07	This study
sCT05	*C. testosteroni KF-1* Δ*pmdD*::0 + pCT08	This study
sCT06	*C. testosteroni KF-1* Δ*pmdD*::0 Δ*pmdK*::0	This study

### Shake flask PDC production experiments

Single colonies of engineered strains were cultivated overnight at 30 °C while shaking (200 rpm) in 5 mL of selective LB medium. These overnight seed cultures were then diluted 200-fold in 100 mL fresh, preheated minimal medium in a 500 mL shake flask containing both glycolic acid and TPA as carbon sources. After 24 h of incubation (30 °C, 200 rpm), samples were taken to evaluate PDC production and TPA consumption. Samples were centrifuged to remove cells and the supernatant was stored at -20 °C till further analysis.

### Monitoring of TPA and glycolic acid co-consumption

To monitor the co-consumption of TPA and glycolic acid, the same procedure was used as described above. However, sampling occurred more frequently.

### Determination of promoter activity

Promoter activities were determined by cloning the upstream region of the corresponding operon in front of a fluorescent protein. After transformation and confirmation of plasmid sequence and plasmid presence, single colonies were grown in a sterile 96-well flat-bottomed microtiter plate (Greiner Bio-One) enclosed by a Breath-Easy® sealing membrane containing 150 µL selective LB medium and incubated on a Compact Digital Microplate Shaker (ThermoFisher Scientific, 3 mm orbit) at 800 rpm and 30 °C for 24 h. Subsequently, strains were diluted 150-fold in selective minimal medium containing glycolic acid as carbon source. To induce fluorescence 2 g/L TPA was added. To measure growth and fluorescence output, the different cultures were grown in a sterile 96-well flat-bottomed, black microtiter plate (Greiner Bio-One) using the same sealing membrane as above using a Tecan Infinite® M200Pro operated at 30 °C. Optical densities were measured at 600 nm and fluorescence of mCherry occurred with excitation at 575 nm and emission at 620 nm. Measurement were taken at regular intervals. Finally, once the cultures reached the stationary phase, fluorescence was plotted in function of optical density and linear regression was performed to the data corresponding to the exponential growth phase. The slope of this linear regression was used as a measure for promoter activity.

### Bioreactor production experiment

Fed-batch fermentations were conducted using a 4 L double jacket glass vessel controlled by a Biostat B + control unit (Sartorius Stedim Biotech, Germany). The initial batch volume consisted of 2.5 L minimal medium as described above with the sole difference that TPA was added to a concentration of 15 mM. An overnight preculture grown on the same minimal medium was used to inoculate (10%) the bioreactor. The fermentation culture was maintained at 30 °C and 1 M of KOH and 500 g/L glycolic acid were used to maintain the pH at 7. To the latter (NH_4_)_2_SO_4_ was added to a C:N-ratio of 10. The gas flow was fixed at 1.5 L/min and the agitation ranged from 100 to 700 rpm as function of the dissolved oxygen (kept above 20%). Off-gas analysis (EGAS-1, Sartorius AG, Germany) provided on-line measurement of CO_2_ and O_2_. All parameters were monitored on the control unit itself. Finally, a 20 g/L solution of TPA adjusted to pH 7 was used as a feed for the addition of TPA to the culture broth. The feed was started after 18.5 h and added around 1 g of TPA per hour. Samples were taken at regular intervals to monitor substrate consumption, product formation and growth of the strain.

### Analytical methods

PDC and TPA were quantified using a Shimadzu Nexera X2 UHPLC device connected to a photodiode array detector scanning between 190 and 390 nm. Metabolites were separated using a Phenomenex Rezex™ ROA-Organic Acid H+ (8%) column operated at 60 °C. The mobile phase consisted of 5 mM H_2_SO_4_ at a flow rate of 0.3 mL.min^− 1^. Products were identified by comparing the retention times and spectral profiles with pure compounds and were calculated based on a calibration curve generated for each compound. In the case of PDC, a self-purified standard was used. To quantify product titers, the peak area at 254 nm was used in both cases.

### PDC purification and structural analysis

PDC was purified based on already described methods [20]. In short, the culture broth was first centrifuged to remove the bacterial cells. Subsequently, the broth was concentrated using a rotary evaporator (Büchi Rotavapor R-134 and Waterbath B-480). Once concentrated, the solution was acidified to a pH < 2 with sulphuric acid to precipitate PDC. After precipitation, the molecules were washed 3 times with distilled water to remove excess salts and centrifuged in between to separate liquids from solids. Finally, the precipitate was dried overnight in a 70 °C oven to remove the remaining water. Purity of the isolated PDC was analysed with a Phenomenex Rezex™ ROA-Organic Acid H+ (8%) column operated at 60 °C using a Shimadzu Nexera X2 UHPLC device connected to a photodiode array detector scanning between 190 and 390 nm and also connected to a LCMS-2020 mass spectrometer (Shimadzu). The mobile phase consisted of 0.1% frmic acid at a flow rate of 0.3 mL.min-1. ^1^ H NMR and ^13^ C NMR spectra were recorded at 400 MHz and 100 MHz, respectively (Bruker Avance III-400) in deuterated solvents and compared to published spectra of PDC.

### Statistical analysis

Unless otherwise stated, all statistical calculations were performed using the Jupyter Notebook environment in Anaconda Navigator 2.1.1, all experiments were performed in triplicate, and error bars represent the standard error of the mean. Welch two-sample T-tests were performed for comparing samples. Normality was checked with the Shapiro-Wilk’s Test and homoscedasticity with the Levene’s Test for which the ‘center’ parameter was set to ‘mean’. In case of heteroscedasticity, the Mann-Whitney U Test was used. In all cases, a significance level of 0.05 was applied.

### Electronic supplementary material

Below is the link to the electronic supplementary material.

Supplementary Material 1

## Data Availability

The datasets used and/or analysed during the current study are available from the corresponding author on reasonable request.

## Abbreviations

PCA: Protocatechuic acid
PDC: 2-pyrone-4,6-dicarboxylic acid
TPA: Terephthalic acid

## References

[1] Borrelle SB, Ringma J, Lavender Law K, Monnahan CC, Lebreton L, McGivern A (2020). Predicted growth in plastic waste exceeds efforts to mitigate plastic pollution. Science.

[2] Geyer R. Production, use, and fate of synthetic polymers. Plastic Waste and Recycling. Elsevier; 2020. 13–32.

[3] Verschoor JA, Kusumawardhani H, Ram AFJ, de Winde JH. Toward Microbial Recycling and Upcycling of Plastics: Prospects and Challenges. Vol. 13, Frontiers in Microbiology. 2022. 10.3389/fmicb.2022.821629 PMC8985596 35401461

[4] Zhu B, Wang D, Wei N. Enzyme discovery and engineering for sustainable plastic recycling. Trends in Biotechnology. Volume 40. Elsevier Ltd; 2022. pp. 22–37. 10.1016/j.tibtech.2021.02.008 33676748

[5] Mohanan N, Montazer Z, Sharma PK, Levin DB (2020). Microbial and enzymatic degradation of Synthetic Plastics. Front Microbiol.

[6] Kang MJ, Kim HT, Lee MW, Kim KA, Khang TU, Song HM (2020). A chemo-microbial hybrid process for the production of 2-pyrone-4,6-dicarboxylic acid as a promising bioplastic monomer from PET waste. Green Chem.

[7] Welsing G, Wolter B, Hintzen HMT, Tiso T, Blank LM (2021). Upcycling of hydrolyzed PET by microbial conversion to a fatty acid derivative. Methods Enzym.

[8] Tiso T, Narancic T, Wei R, Pollet E, Beagan N, Schröder K (2021). Towards bio-upcycling of polyethylene terephthalate. Metab Eng.

[9] Kim HT, Kim JK, Cha HG, Kang MJ, Lee HS, Khang TU (2019). Biological valorization of poly(ethylene terephthalate) monomers for Upcycling Waste PET. ACS Sustain Chem Eng.

[10] Kim HT, Hee Ryu M, Jung YJ, Lim S, Song HM, Park J (2021). Chemo-Biological Upcycling of Poly(ethylene terephthalate) to multifunctional coating materials. Chemsuschem.

[11] Kenny ST, Runic JN, Kaminsky W, Woods T, Babu RP, Keely CM (2008). Up-cycling of PET (polyethylene terephthalate) to the biodegradable plastic PHA (polyhydroxyalkanoate). Environ Sci Technol.

[12] Lee S, Lee YR, Kim SJ, Lee JS, Min K (2023). Recent advances and challenges in the biotechnological upcycling of plastic wastes for constructing a circular bioeconomy. Chem Eng J.

[13] Masai E, Shinohara S, Hara H, Nishikawa S, Katayama Y, Fukuda M (1999). Genetic and biochemical characterization of a 2-Pyrone-4,6-Dicarboxylic acid hydrolase involved in the Protocatechuate 4,5-Cleavage pathway of Sphingomonas paucimobilis SYK-6. J Bacteriol.

[14] Qian Y, Otsuka Y, Sonoki T, Mukhopadhyay B, Nakamura M, Jellison J et al. Engineered microbial production of 2-pyrone-4,6-dicarboxylic acid from lignin residues for use as an industrial platform chemical. BioResources. 2016;11.

[15] Lee S, Jung YJ, Park SJ, Ryu MH, Kim JE, Song HM et al. Microbial production of 2-pyrone-4,6-dicarboxylic acid from lignin derivatives in an engineered Pseudomonas putida and its application for the synthesis of bio-based polyester. Bioresour Technol. 2022;352. 10.1016/j.biortech.2022.127106 35378283

[16] Michinobu T, Hishida M, Sato M, Katayama Y, Masai E, Nakamura M (2008). Polyesters of 2-pyrone-4,6-dicarboxylic acid (PDC) obtained from a metabolic intermediate of lignin. Polym J.

[17] Mlchinobu T, Bito M, Amada YY, Tanimura M, Katayama Y, Masai E et al. Fusible, elastic, and biodegradable polyesters of 2-pyrone-4, 6-dicarboxylic acid (PDC). Polym J:1111–6.

[18] Bito M, Otsuka Y, Nakamura M, Masai E, Katayama Y, Shigehara K (2019). Unique complexation behavior of Alkali Metal Ions and 2-Pyrone-4,6-Dicarboxylic acid (PDC) obtained from a metabolic Intermediate of Lignin. Waste Biomass Valorization.

[19] Shikinaka K, Otsuka Y, Iguchi Y, Nakamura M, Itoh Y, Masai E (2016). Preferential cesium ion trapping by 2-pyrone-4,6-dicarboxylic acid (PDC) obtained from a metabolic intermediate of lignin, a woody biomass resource. J Nucl Sci Technol.

[20] Johnson CW, Salvachúa D, Rorrer NA, Black BA, Vardon DR et al. St. John PC,. Innovative Chemicals and Materials from Bacterial Aromatic Catabolic Pathways. Joule. 2019;3(6):1523–37.

[21] Otsuka Y, Nakamura M, Shigehara K, Sugimura K, Masai E, Ohara S (2006). Efficient production of 2-pyrone 4,6-dicarboxylic acid as a novel polymer-based material from protocatechuate by microbial function. Appl Microbiol Biotechnol.

[22] Otsuka Y, Araki T, Suzuki Y, Nakamura M, Kamimura N, Masai E. High-level production of 2-pyrone-4,6-dicarboxylic acid from vanillic acid as a lignin-related aromatic compound by metabolically engineered fermentation to realize industrial valorization processes of lignin. Bioresour Technol. 2023;128956. 10.1016/j.biortech.2023.128956 36965585

[23] Suzuki Y, Okamura-Abe Y, Nakamura M, Otsuka Y, Araki T, Otsuka H (2020). Development of the production of 2-pyrone-4,6-dicarboxylic acid from lignin extracts, which are industrially formed as by-products, as raw materials. J Biosci Bioeng.

[24] Luo ZW, Kim WJ, Lee SY (2018). Metabolic Engineering of Escherichia coli for efficient production of 2-Pyrone-4,6-dicarboxylic acid from glucose. ACS Synth Biol.

[25] Nakajima M, Nishino Y, Tamura M, Mase K, Masai E, Otsuka Y (2009). Microbial conversion of glucose to a novel chemical building block, 2-pyrone-4,6-dicarboxylic acid. Metab Eng.

[26] Weiss M, Kesberg AI, Labutti KM, Pitluck S, Bruce D, Hauser L (2013). Permanent draft genome sequence of Comamonas testosteroni KF-1. Stand Genomic Sci.

[27] Michinobu T, Bito M, Yamada Y, Katayama Y, Noguchi K, Masai E (2007). Molecular Properties of 2-Pyrone-4,6-dicarboxylic acid (PDC) as a stable metabolic Intermediate of Lignin isolated by Fractional Precipitation with na + ion. B Chem Soc Jpn.

[28] Hosaka M, Kamimura N, Toribami S, Mori K, Kasai D, Fukuda M (2013). Novel tripartite aromatic acid transporter essential for terephthalate uptake in Comamonas sp. strain E6. Appl Environ Microbiol.

[29] Kamimura N, Aoyama T, Yoshida R, Takahashi K, Kasai D, Abe T (2010). Characterization of the protocatechuate 4,5-cleavage pathway operon in comamonas sp. strain e6 and discovery of a novel pathway gene. Appl Environ Microbiol.

[30] Wilkes RA, Waldbauer J, Caroll A, Nieto-Domínguez M, Parker DJ, Zhang L et al. Complex regulation in a Comamonas platform for diverse aromatic carbon metabolism. Nat Chem Biol. 2023. 10.1038/s41589-022-01237-7 PMC10154247 36747056

[31] Vlaeminck E, Quataert K, Uitterhaegen E, De Winter K, Soetaert WK (2022). Advanced PHB fermentation strategies with CO2-derived organic acids. J Biotechnol.

[32] Maung NW, Smolke CD (2007). A modular and extensible RNA-based gene-regulatory platform for engineering cellular function. Proc Natl Acad Sci U S A.

[33] Kovach ME, Elzer PH, Steven Hill D, Robertson GT, Farris MA, Roop RM (1995). Four new derivatives of the broad-host-range cloning vector pBBR1MCS, carrying different antibiotic-resistance cassettes. Gene.

[34] Tang Q, Lu T, Liu SJ (2018). Developing a Synthetic Biology Toolkit for Comamonas testosteroni, an Emerging Cellular Chassis for Bioremediation. ACS Synth Biol.

