# Supplementary material for: Engineering Comamonas testosteroni for the production of 2-pyrone-4,6-dicarboxylic acid as a promising building block
Source: Microb Cell Fact. 2023 Sep 19;22:188. doi: 10.1186/s12934-023-02202-2 (PMC10510227; doi:10.1186/s12934-023-02202-2)
Supplement: Supplementary file 1 — Supplementary Material 1 [file 12934_2023_2202_MOESM1_ESM.docx]

**Supplementary files**

**Table S.1: Trace element added to different minimal media and their final concentration.**

| **Component** | **Final concentration (mg/L)** |
| --- | --- |
| FeCl_2_.4H_2_O | 3.6 |
| CaCl_2_.2H_2_O | 5 |
| MnCl_2_.H_2_O | 1.3 |
| CuCl_2_.2H_2_O | 0.38 |
| CoCl_2_.6H_2_O | 0.5 |
| ZnCl_2_ | 0.94 |
| H_3_BO_4_ | 0.0311 |
| Na_2_EDTA.2H_2_O | 0.4 |
| Thiamine.HCl | 1.01 |
| Na_2_MoO_4_.2H_2_O | 0.0967 |


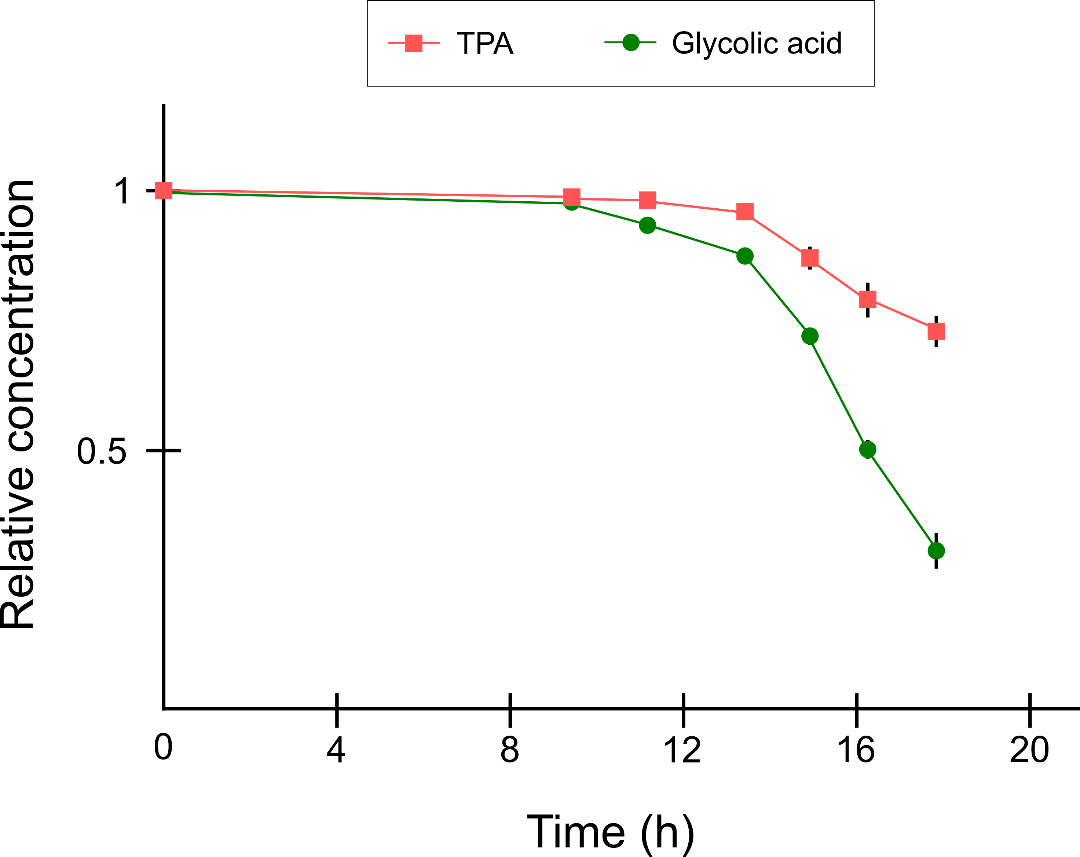


**Figure S.1: Consumption of TPA and glycolic acid by the wild-type strain. No color in print.**


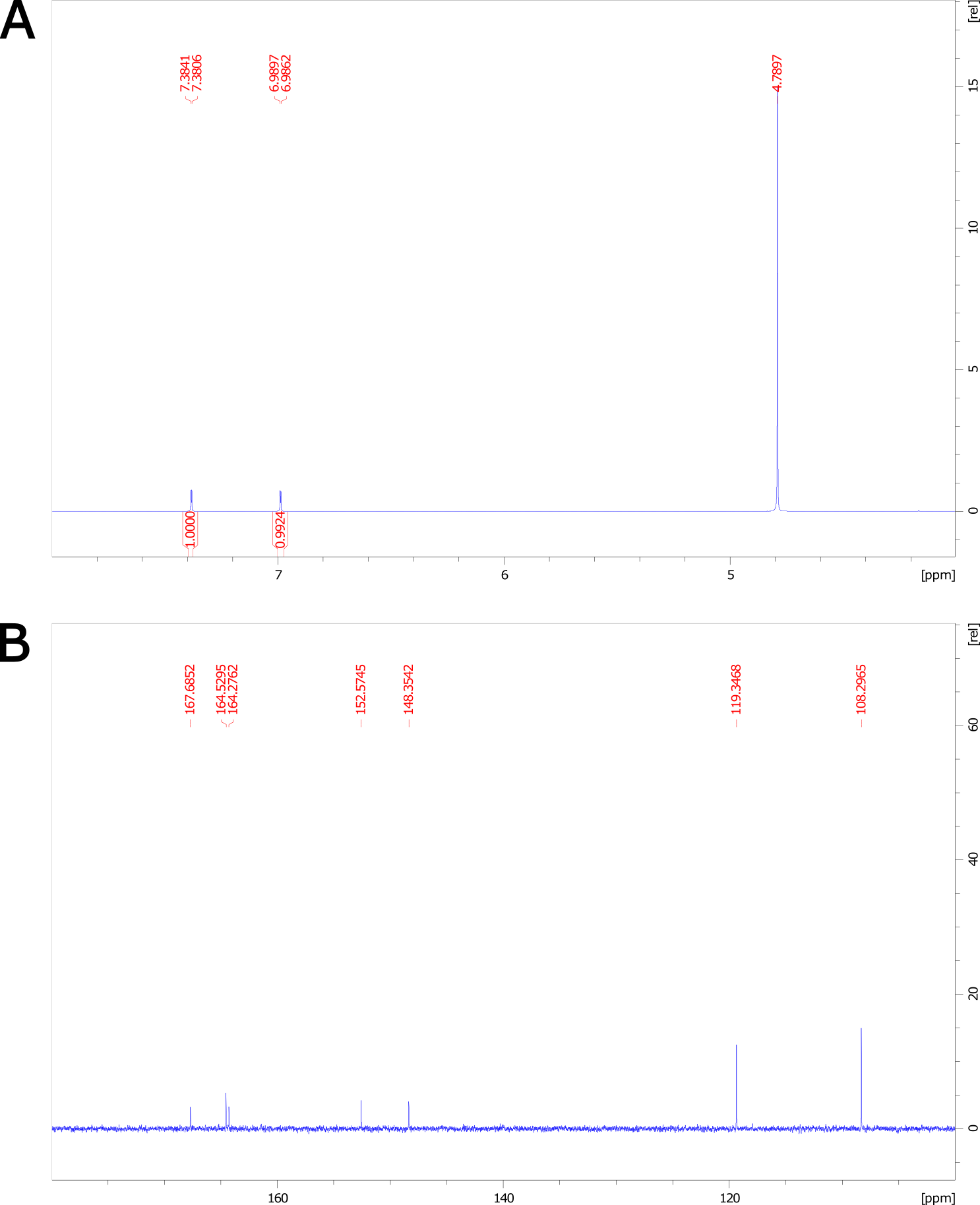


**Figure S.2**: **NMR spectra of the obtained product. Both the ^1^H NMR (400 MHz, D_2_O): δ 6.99 (1H, d, *J* = 1.4 Hz); 7.38 (1H, d, *J* = 1.4 Hz) (part A) and the ^13^C NMR (100 MHz, D_2_O): δ 108.3 (CH); 119.3 (CH); 148.4 (C_q_); 152.6 (C_q_); 164.3 (C=O); 164.5 (C=O); 167.7 (C=O) (part B) correspond to PDC as a target molecule. No color in print**
